# Inhibitory Effect of the LY2109761 on the Development of Human Keloid Fibroblasts

**DOI:** 10.1155/2021/8883427

**Published:** 2021-02-10

**Authors:** Xiuxia Wang, Chuan Gu, Feng Shang, Rui Jin, Jia Zhou, Zhen Gao

**Affiliations:** ^1^Department of Plastic and Reconstructive Surgery, Ninth People's Hospital, School of Medicine, Shanghai Jiao Tong University, Shanghai 200011, China; ^2^Department of Burns, Second Affiliated Hospital of Shandong First Medical University, Taian, China

## Abstract

Keloids are scars characterized by abnormal proliferation of fibroblasts and overproduction of extracellular matrix components including collagen. We previously showed that LY2109761, a transforming growth factor- (TGF-) *β* receptor inhibitor, suppressed the secretion of matrix components and slowed the proliferation of fibroblasts derived from human hypertrophic scar tissue. However, the exact mechanism underlying this effect remains unclear. Here, we replicated the above results in keloid-derived fibroblasts and show that LY2109761 promoted apoptosis, decreased the phosphorylation of Smad2 and Smad3, and suppressed TGF-*β*1. These results suggest that the development and pathogenesis of keloids are positively regulated by the Smad2/3 signaling pathway and the upregulation of TGF-*β*1 receptors. LY2109761 and other inhibitors of these processes may therefore serve as therapeutic targets to limit excessive scarring after injury.

## 1. Introduction

Keloids represent a dysregulated cutaneous wound healing process characterized by abnormal proliferation of fibroblasts and excessive deposition of collagen [1, 2] and can form after burns or other skin injuries [3, 4]. Although keloids share several features with hypertrophic scars, such as excessive collagen deposition, they present also distinct features: they can extend beyond the borders of the wound, rarely regress over time, and have a high rate of recurrence [5, 6]. At present, the exact mechanisms underlying the formation and progression of keloids remain unclear.

Surgical treatment often results in the recurrence of keloids, which can be larger than the original lesion. Radiation, which is currently used as a postoperative adjuvant therapy and has shown significant efficacy in reducing recurrence, is associated with an increased risk of skin cancer [5]. Other treatments including laser therapy, cryotherapy, and occlusive dressings have shown limited efficacy and significant adverse effects.

Keloid fibroblasts are the primary cells involved in the formation of the wound healing scar, and their elevated proliferation and limited apoptosis lead to excess scarring [7–9]. LY2109761, a transforming growth factor- (TGF-) *β* receptor inhibitor, reduces tumor cell growth, intravasation, and the metastatic dissemination of hepatocellular carcinoma (HCC) cells through different molecular mechanisms [10]. We previously showed that LY2109761 inhibited human hypertrophic scar formation [11]; however, its effect on keloid formation has not been tested yet.

In a separate study, we showed that growth differentiation factor- (GDF-) 9 promoted fibroblast proliferation and migration in keloids via the Smad2/3 pathway [5], while others have demonstrated that TGF-*β*/Smad signaling plays an essential role in the development of keloids [12–15]. In the present study, we focused on changes in the Smad2/3 signaling pathway in response to LY2109761 treatment. Specifically, we isolated keloid-derived fibroblasts and treated them with two concentrations of LY2109761 (5 and 10 *μ*M). We assessed the effect of LY2109761 on keloid-derived fibroblast proliferation and apoptosis, suppression of the TGF-*β*/Smad signaling pathway, and expression of key factors involved in keloid formation.

## 2. Materials and Methods

### 2.1. Human Subjects

Surgically excised keloids (*n* = 6) were donated from patients, who underwent plastic surgery without previous treatment for scarring (Table 1). The handling of human tissue was approved by the Ethics Committee of Shanghai Ninth People's Hospital (Shanghai, China). Detailed information on the patients, lesion locations, and harvested areas is provided in Table 1.

### 2.2. Primary Cell Isolation and In Vitro Culture

Fibroblasts derived from excised keloids were isolated as previously described with several modifications [12]. Briefly, keloids were immersed in 2.5% chloramphenicol solution, rinsed with phosphate-buffered saline (PBS), cut into small pieces (approximately 24 mm^2^), and immersed in 0.2% dispase (Roche Diagnostics, Indianapolis, IN, USA) dissolved in Dulbecco's modified Eagle medium (DMEM; Gibco, Gaithersburg, MD, USA) containing 10% fetal bovine serum (FBS). Keloids were incubated overnight at 4°C to enzymatically dissociate scar tissue. The next day, the dermis was mechanically isolated from the surrounding scar tissue with forceps and subjected to another round of enzymatic digestion with 0.2% collagenase II (SERVA, Heidelberg, Germany) in DMEM for 3 h at 37°C on a rotator. The digested cell suspension was filtered through a nylon mesh; washed in DMEM containing 10% FBS, penicillin (100 U/mL), and streptomycin (0.1 mg/mL); and centrifuged at 524 × g for 5 min. Pelleted fibroblasts were resuspended in DMEM supplemented with 10% FBS, seeded in 10 cm culture dishes (BD, Falcon, TX, USA) and cultured at 37°C with 5% CO_2_ until they reached 80% confluence. Confluent cells were then subcultured at a ratio of 1 : 3. All subsequent experiments were performed using mixed fibroblasts extracted from human keloid samples mentioned above.

### 2.3. Scratch Wound Healing Assay

A scratch wound assay was used to evaluate cell migration as previously described [16]. Briefly, fibroblasts were seeded in 6-well plates coated with collagen (BD Biosciences, San Diego, CA, USA) and cultured until they reached 80% confluence. Cells were then treated with LY2109761 (dissolved in DMSO; Sigma-Aldrich, St. Louis, MO, USA) at 5 or 10 *μ*M for 36 h. Control fibroblasts were treated with DMSO only. A pipette tip was used to make a “wound” of approximately 0.45–0.50 mm in width at a 90° angle to the bottom of the dish. Cell migration into the wound area was assessed immediately after wound generation and at 24 and 48 h postwound generation using a light microscope (Nikon, Tokyo, Japan). The percentage of cells migrating into the wound area was calculated. Three independent fields were analyzed in each well, and each experiment was performed in triplicate.

### 2.4. Enzyme-Linked Immunosorbent Assay (ELISA)

Keloid-derived fibroblasts were incubated with LY2109761 (5 or 10 *μ*M) or vehicle control (DMSO) for 48 h. After treatment, the supernatant was collected and the concentration of TGF-*β* was measured using a human TGF-*β* ELISA kit (R&D, San Jose, CA, USA). Absorbance was measured at 450 nm using a microreader (Thermo Fisher Scientific, Waltham, MA, USA). The assay was performed in triplicate and repeated in three cell samples.

### 2.5. Quantitative Real-Time PCR (qRT-PCR)

Total RNA was extracted from cultured fibroblasts as previously described [5]. Briefly, cells were incubated with LY2109761 (5 or 10 *μ*M) or vehicle control (DMSO) for 48 h, after which total RNA was extracted using the TRIzol reagent (Invitrogen, Carlsbad, CA, USA) and reverse-transcribed using the AMV Reverse Transcription System according to the manufacturer's instructions (TaKaRa Bio, Shiga, Japan). qRT-PCR was performed using SYBR Green PCR mix (Applied Biosystems, Foster City, CA, USA) on an ABI Prism 7900HT thermocycler (Applied Biosystems) under the following conditions: 95°C for 10 min followed by a two-step PCR program including 95°C for 15 s and 60°C for 1 min for 40 cycles. The amplified products were normalized to glyceraldehyde 3-phosphate dehydrogenase (GAPDH). PCR primers are listed in Table 2. Each assay was performed in triplicate.

### 2.6. Western Blot Analysis

Total protein was extracted from cells treated with various LY2109761 concentrations as described above. Briefly, 50 *μ*g of total protein was separated by 10% SDS-PAGE and transferred to a polyvinylidene difluoride membrane (Millipore, Bedford, MA, USA). The membrane was then incubated overnight at 4°C with primary antibodies diluted in 1% nonfat dried milk (Sigma-Aldrich) plus 0.1% Tween. Antibodies targeting collagen I, collagen III, fibronectin, matrix metalloprotease- (MMP-) 1, MMP-3, TGF-*β*1, Smad2, phospho- (p-) Smad2, Smad3, p-Smad3, and GAPDH (internal control) were used (Abcam, Cambridge, UK). Next, the membrane was incubated with the appropriate horseradish peroxidase-conjugated secondary antibodies for 2 h at room temperature. Membrane-bound proteins were detected using enhanced chemiluminescence detection kit reagents (Pierce, Rockford, IL, USA). Semiquantitative analysis (AlphaView Software 3.3; ProteinSimple, San Jose, CA, USA) results are expressed as the optical density (OD × mm^2^) normalized to GADPH. Relative protein expression was determined by ImageJ software (NIH, Bethesda, MD, USA).

### 2.7. Cell Proliferation Assays

The proliferation of LY210971-treated cells (5 or 10 *μ*M) and control cells was assessed using the cell counting kit-8 (CCK-8 kit; Dojindo, Kumamoto, Japan) according to the manufacturer's instructions. Briefly, cells were seeded in 96-well plates at a density of 2 × 10^4^ cells/well. They were cultured for 1, 3, 5, 7, and 9 h before being analyzed. After each time point, 10 *μ*L of CKK-8 was added to each well and the plate was incubated for 4 h at 37°C. Absorbance was then read using a microplate reader (Molecular Devices, Sunnyvale, CA, USA) at 450 nm. Each sample group contained six parallel wells and was repeated in triplicate.

### 2.8. Flow Cytometric Analysis of Apoptosis

Cells treated with 5 or 10 *μ*M LY2109761 were assessed with a cell apoptosis kit containing Annexin V-FITC and propidium iodide (PI; eBioscience San Diego, CA, USA) according to the manufacturer's instructions. Briefly, cells were washed twice in PBS and adjusted to 1 × 10^6^ cells/mL with 1× Annexin-binding buffer (eBioscience). Annexin V-FITC (10 *μ*L) and PI (10 *μ*L) were added to a 100 *μ*L cell suspension and incubated for 15 min at room temperature. Stained cells were then analyzed using multiple-color flow cytometry on a FACS Aria cell sorter (BD Biosciences).

### 2.9. Statistical Analysis

All data are presented as the mean ± standard error of the mean (SEM). Statistical significance was measured using Student's *t*-test for differences between two groups or by one-way ANOVA for multiple group comparisons. *P* < 0.05 was considered statistically significant. All statistical analyses were performed using SPSS version 13.0 (SPSS Inc., Chicago, IL, USA).

## 3. Results

### 3.1. LY2109761 Inhibits the Expression of Keloid-Related Factors

**Figure 1:**
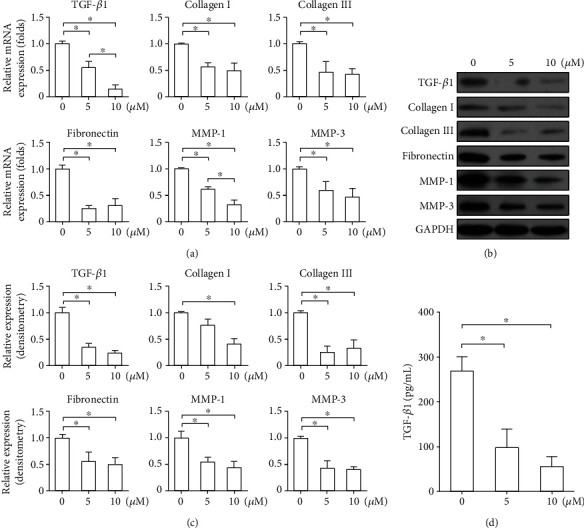
LY2109761 inhibits the expression of keloid-derived fibroblast-related factors in vitro. (a) Relative mRNA expression levels of transforming growth factor (TGF)-*β*1, collagens I and III, fibronectin, and matrix metalloprotease- (MMP-) 1, and MMP-3 as determined by qRT-PCR. (b) Representative western blots showing protein expression of the above gene products. (c) Relative protein expression. (d) ELISA results showing TGF-*β*1 secretion in response to increasing concentrations of LY2109761. Glyceraldehyde 3-phosphate dehydrogenase (GADPH) was used as the internal control. ^∗^*P* < 0.05 between LY2109761 groups and DMSO control; ^∗∗^*P* < 0.05 between LY2109761 5*μ*M and 10 *μ*M treatment groups.

The effect of LY2109761 on fibroblasts isolated from keloid tissue was assessed by qRT-PCR, western blotting, and ELISA. LY2109761 treatment at 5 and 10 *μ*M significantly inhibited the mRNA and protein expression of key keloid-related factors, including TGF-*β* (Figures 1(a)–1(d)), collagen I (10 *μ*M only; Figures 1(a)–1(c)), collagen III (Figures 1(a)–1(c)), fibronectin (Figures 1(a)–1(c)), MMP-1 (Figures 1(a)–1(c)), and MMP-3 (Figures 1(a)–1(c)) compared with control treatment (*P* > 0.05).

### 3.2. LY2109761 Inhibits Keloid-Derived Fibroblast Proliferation

**Figure 2:**
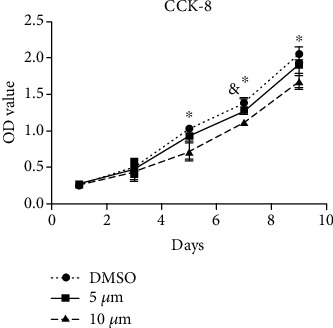
LY2109761 inhibits keloid-derived fibroblast proliferation. Cell proliferation was assessed using the CCK-8 assay in keloid-derived fibroblasts treated with increasing concentrations of LY2109761 on days 3, 5, 7, and 9. Data are presented as the mean ± SEM from three independent experiments. ^∗^*P* < 0.05 between LY2109761 groups and DMSO control; ^∗∗^*P* < 0.05 between LY2109761 5 *μ*M and 10 *μ*M treatment groups.

The CCK-8 assay was used to evaluate the effect of LY2109761 on the proliferation of keloid-derived fibroblasts. Results showed that LY2109761 (5 and 10 *μ*M) significantly inhibited the proliferation of fibroblasts from days 5 to 9 compared with the control treatment (*P* < 0.05; Figure 2).

### 3.3. LY2109761 Inhibits Keloid-Derived Fibroblast Migration and Invasion

**Figure 3:**
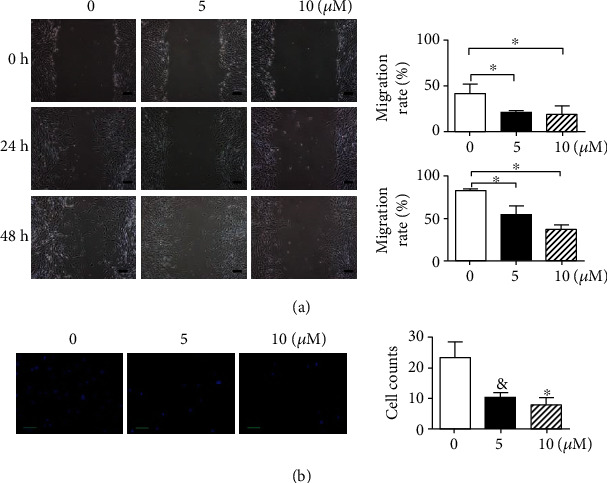
LY2109761 inhibits keloid-derived fibroblast migration and invasion. (a) Scratch experiments using increasing doses of LY2109761; representative images are shown on the left, and summarized results are shown on the right as the mean ± SEM from three independent experiments. (b) Invasion experiments at increasing doses of LY2109761; representative images are shown on the left, and summarized results are shown on the right as the mean ± SEM from three independent experiments. Scale bar = 50 *μ*m. ^∗^*P* < 0.05 between LY2109761 groups and DMSO control; ^∗∗^*P* < 0.05 between LY2109761 5 *μ*M and 10 *μ*M treatment groups.

As shown in Figure 3, treatment with LY2109761 significantly inhibited keloid-derived fibroblast migration into the scratched area at 24 and 48 h compared with control-treated fibroblasts (*P* < 0.05). At 24 h, almost 41.38 ± 5.776% of keloid-derived fibroblasts migrated into the wound, whereas only 21% of fibroblasts did so in the two LY2019761 groups. By 48 h, 83.37 ± 0.7987% of fibroblasts in the control group migrated into the wound, which was significantly more than the percentage observed in the groups treated with 5 *μ*M (54.32 ± 5.904%) and 10 *μ*M (36.73 ± 3.227%) LY2019761.

### 3.4. LY2109761 Promotes Keloid-Derived Fibroblast Apoptosis

**Figure 4:**
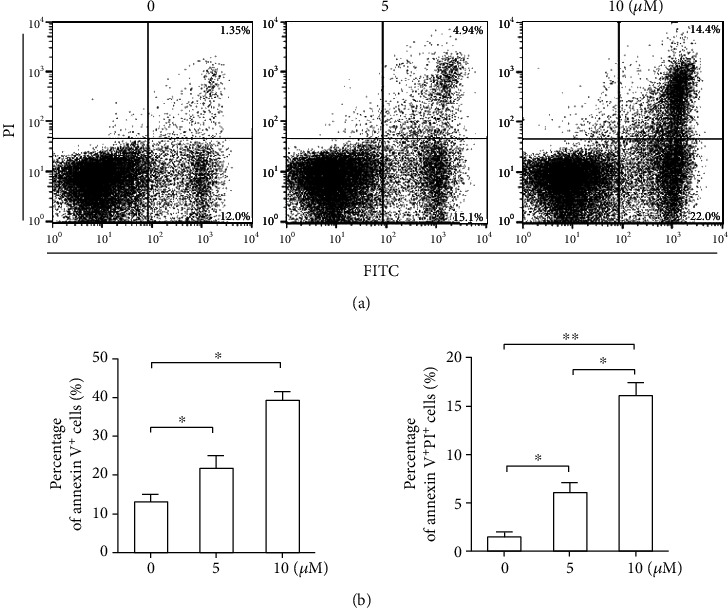
LY2109761 promotes keloid-derived fibroblast apoptosis. Annexin V-FITC/PI staining was followed by treatment with increasing doses of LY2109761. (a) Representative scatter plot images. (b) Summarized results are shown as the mean ± SEM from three independent experiments. ^∗^*P* < 0.05 between LY2109761 groups and DMSO control; ^∗∗^P < 0.05 between LY2109761 5 *μ*M and 10 *μ*M treatment groups.

Annexin V-FITC/PI staining and fluorescent cell sorting were used to evaluate the effect of LY2109761 on fibroblast apoptosis. LY2109761 treatment significantly increased apoptosis in a dose-dependent manner compared with control treatment (*P* < 0.05; Figure 4). As the concentration increased, inhibition was enhanced, too, reaching more than 90%.

### 3.5. LY2109761 Suppresses the Smad2/Smad3 Signaling Pathway

**Figure 5:**
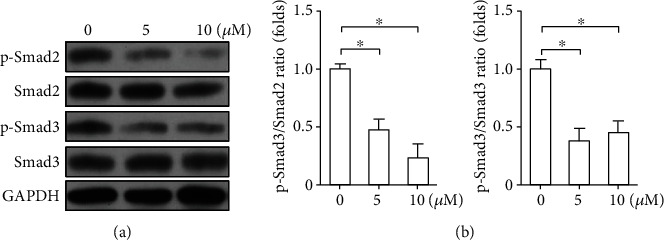
LY2109761 inhibits the Smad2/Smad3 signaling pathway. Keloid-derived fibroblasts were treated with increasing doses of LY2109761. (a) Representative western blots showing protein expression of Smad2 and Smad3 and their respective phosphorylated forms (p-Smad2 and p-Smad3). (b) Relative protein expression levels are shown as the mean ± SEM from three independent experiments. Glyceraldehyde 3-phosphate dehydrogenase (GADPH) was used as an internal control. ^∗^*P* < 0.05 between LY2109761 groups and DMSO control; ^∗∗^*P* < 0.05 between LY2109761 5 *μ*M and 10 *μ*M treatment groups.

To identify the signaling pathway involved in LY2109761-induced keloid fibroblast alterations, protein expression of Smad2 and Smad3, as well as that of their respective phosphorylated forms (p-Smad2 and p-Smad3), was assessed by western blotting. As shown in Figure 5, treatment with LY2109761 at increasing doses significantly downregulated the phosphorylated forms of Smad2 and Smad3 compared with control treatment (*P* < 0.05).

### 3.6. LY2109761 Upregulates the mRNA and Protein Expression of GDF-9

**Figure 6:**
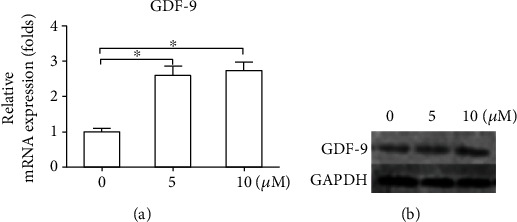
LY2109761 upregulates the expression of GDF-9. Keloid-derived fibroblasts were treated with increasing doses of LY2109761. (a) Relative mRNA expression levels of GDF-9 as determined by qRT-PCR. (b) Representative western blots showing protein expression of GDF-9. ^∗^*P* < 0.05 between LY2109761 groups and DMSO control; ^∗∗^*P* < 0.05 between LY2109761 5 *μ*M and 10 *μ*M treatment groups.

To identify the effect of LY2109761the expression of GDF-9 of keloid fibroblast, mRNA and protein of GDF-9 were assessed. As shown in Figure 6, treatment with LY2109761 at increasing doses significantly upregulated the mRNA and protein expression of GDF-9 compared with control treatment (*P* < 0.05).

## 4. Discussion

The present study investigated the effect and the underlying mechanisms of LY2109761 treatment on keloid-derived fibroblasts. LY2109761 treatment at two concentrations (5 and 10 *μ*M) inhibited the expression of key extracellular matrix factors secreted by fibroblasts and responsible for promoting keloid development. This included TGF-*β*, collagens I and III, fibronectin, and MMP-1, and MMP-3, at both mRNA and protein levels. Previous studies reported that 10–30 *μ*M peptide inhibitors or LY2109761 were sufficient to exert an inhibitory effect [11, 17, 18]. Here, we show that 5 *μ*M LY2019761 was sufficient to significantly inhibit factors associated with keloid formation, suggesting that low doses may be sufficient to slow or prevent pathological scarring.

LY2109761, a TGF-*β* inhibitor, prevents the release of connective tissue growth factors in tumors, thereby reducing the size and metastasis of tumors [19, 20]. He et al. [10] reported that LY2109761 in combination with transcatheter arterial chemoembolization reduced liver tumor size and metastases in a Smad2-dependent manner in both a rabbit tumor model and an HCC model [10]. A study by Xu et al. [21] showed that LY2109761 reversed the antiapoptotic effect of TGF-*β*1 in myelomonocyte leukemia cells [21]. More recently, our group used human hypertrophic scar-derived fibroblasts and showed that LY2109761 inhibited the secretion of factors related to the development of hypertrophic scars and reduced the migration of the ensuing fibroblasts [5]. Present results are in agreement with these previous observations.

Although conclusive, our previous study did not elucidate the mechanism underlying the effect of LY2109761 treatment. Based on reports showing that LY2109761 inhibited the Smad signaling pathway in human and rabbit tumor models [10], we hypothesized that a similar mechanism was involved in the suppression of keloid-derived fibroblast secretion and proliferation, as well as in the activation of apoptosis. We report that the activated forms of Smad2 and Smad3, p-Smad2 and p-Smad3, were significantly downregulated at 48 h after LY2109761 treatment in a dose-dependent manner. We previously showed that silencing of the TGF-*β* family member GDF-9 in keloid-derived fibroblasts downregulated p-Smad2 and p-Smad3 [5]. However, silencing of GDF-9 had no effect on the expression of TGF-*β*1, whereas in the current study, LY2109761 clearly suppressed TGF-*β*1 expression but upregulated GDF-9 expression. We speculate that the possible reason for this is that GDF-9, as one of the ligands of TGF-*β* receptors, is produced more to antagonize the inhibitory effect of TGF-*β* signaling pathway. In other words, it is a negative feedback mechanism. The overall effect of LY2109761 on keloid fibroblasts is inhibition, even though GDF-9 was partially elevated. Existing evidence indicates that regulation of TGF-*β* receptors is a key factor associated with keloid pathogenesis [22]. TGF-*β*1 is involved in the formation of keloids, and normal fibroblasts produce up to 12-fold higher levels of collagen following treatment with TGF-*β*1 [23, 24]. Another study also showed that interactions between the TGF-*β*/Smad and Wnt/*β*-catenin pathways were associated with the formation of keloids and hypertrophic scars [25].

Here, we provide further evidence that Smad2/3 signaling and the upregulation of TGF-*β* receptors are directly involved in keloid pathogenesis [26, 27]. More importantly, we show that LY2109761, a TGF-*β* receptor inhibitor, negatively regulated the Smad2/3 signaling pathway and downregulated the expression of TGF-*β*1 in keloid-derived fibroblasts. These results suggest that inhibition of pathways/receptors involved in keloid formation and pathogenesis may offer potential therapeutic strategies to limit scarring after injury.

## Figures and Tables

**Table 1:** Demographic data of keloid samples used in this study.

Case	Gender	Age (years)	Origin
1	F	36	Chest
2	F	29	Back
3	F	22	Chest
4	M	41	Arm
5	M	25	Chest
6	M	30	Shoulder

**Table 2:** Quantitative real-time PCR primers.

Name	Sequence
TGF-*β* forward	5′-GAAGTGGATCCACGAGCCCAAG-3′
TGF-*β* reverse	5′-GCTGCACTTGCAGGAGCGCAC-3′
GDF-9 forward	5′-CAGAAGTGACCGTAGTCCACCC-3′
GDF-9 reverse	5′-CACCTCGCCCAACAGATAGAAC-3′
Collagen I forward	5′-GGCGGCCAGGGCTCCGACCC-3′
Collagen I reverse	5′-AATTCCTGGTCTGGGGCACC-3′
Collagen III forward	5′-TGGTGTTGGAGCCGCTGCCA-3′
Collagen III reverse	5′-CTCAGCACTAGAATCTGTCC-3′
Fibronectin forward	5′-GCCACTGGAGTCTTTACCACA-3′
Fibronectin reverse	5′-CCTCGGTGTTGTAAGGTGGA-3′
MMP-1 forward	5′-GCAGCTGTAGATGTCCTTGGGGT-3′
MMP-1 reverse	5′-CCTCGGTGTTGTAAGGTGGA-3′
MMP-3 forward	5′-AGGACAAAGCAGGATCACAGTTG-3′
MMP-3 reverse	5′-CCTGGTACCCACGGAACCT-3′
GAPDH forward	5′-TCACCATCTTCCAGGAGCG-3′
GAPDH reverse	5′-CTGCTTCACCACCTTCTTGA-3′

## Data Availability

The data used to support the findings of this study are included within the article.
